# African Primary Care Research: Quantitative analysis and presentation of results

**DOI:** 10.4102/phcfm.v6i1.646

**Published:** 2014-06-06

**Authors:** Bob Mash, Gboyega A. Ogunbanjo

**Affiliations:** 1 Division of Family Medicine and Primary Care, Stellenbosch University, South Africa; 2 Department of Family Medicine, University of Limpopo, Medunsa Campus, South Africa

## Abstract

This article is part of a series on Primary Care Research Methods. The article describes types of continuous and categorical data, how to capture data in a spreadsheet, how to use descriptive and inferential statistics and, finally, gives advice on how to present the results in text, figures and tables. The article intends to help Master's level students with writing the data analysis section of their research proposal and presenting their results in their final research report.

## Abstrait

**Recherche africaine dans le Domaine des Soins primaires: Analyse quantitative et présentation des résultats**. Cet article fait partie d'une série sur les Méthodes de Recherche dans le Domaine des Soins primaires. L'article décrit les types de données continues et catégoriques, comment saisir les données dans un tableur, comment utiliser les statistiques descriptives et déductives et, finalement, donne des conseils sur la présentation des résultats dans un texte, des figures ou des tableaux. L'article a pour but d'aider les étudiants au niveau de la Maitrise à rédiger l'analyse des données de leur proposition de recherche et à présenter leurs résultats dans leur rapport de recherche final.

## Introduction

This article is part of a series on Primary Care Research and focuses on how to capture, clean and analyse quantitative data, as well as how to present your results. Other articles in the series focus on how to write your research proposal, research report and how to collect quantitative data, typically through a survey with a questionnaire. The series is written to support Master's level research in family medicine and primary care in the African context. Your research proposal should contain an analysis plan that explains how you will capture and analyse your data.

## Types of data

When planning your data collection, for example, choosing or designing a questionnaire, it is important to be aware of the type of quantitative data that you will obtain and how this will be analysed. If you do not intend to analyse the data or if it does not directly contribute to answering your aim and objectives, then you should re-consider whether it is worth collecting at all. The selection of statistical approaches varies depending on the type of data and you should describe the different types you will be using in the data analysis section of your research proposal. The following broad types of data exist:^1, 2^

*Continuous data:* This is when the numbers are on a continuous scale, such as weight, age or blood pressure. In other words, they are data that can be measured on a scale and compared with other data. Whenever possible, data should be collected as a continuous variable, rather than as a categorical variable, as you have more statistical power and flexibility during the analysis. For example, it is better to collect the actual age rather than asking people to select which age category they fall within. In addition, it makes it easier to determine the mean, mode, median or standard deviation when the actual ages are collected. Some continuous datasets are distributed normally (with a symmetrical bell-shaped curve when plotted on a graph) and others are not distributed normally (asymmetrical bell-shaped curve when plotted on a graph). Whether the distribution is normal or not makes a difference to the selection of statistical approaches.

*Categorical data:* These are numeric data, which represent different categories or a set of data sorted or divided into different categories, according to the attributes thereof:

- Nominal data: used for categories that are mutually exclusive, but without any specific order or progression in size. Binary data is when there are just two categories such as (yes) and (no), or (male) and (female). Other nominal data categories could be marital status or employment where there are multiple options.
- Ordinal data: when the categories have some kind of order or relationship to each other, such as on a Likert scale or with a progression of income categories.

## Capturing quantitative data

Data are often captured on an Excel^®^ spreadsheet which can then be imported into a statistical software package for analysis. When capturing data, each row is usually a patient or data source and each column a variable. The first row can be used to give each column a brief one word title that will be used in the analysis to refer to that variable. Each column deals with only one variable and the cells should contain either a number (e.g. 1 for male and 2 for female) or a symbol (e.g. M for male and F for female), but should not use both simultaneously. If there are no data available for that cell, it is usual to leave it blank. In a question that has multiple possible responses, each option will need to be treated as a separate column in the spreadsheet. Table 1 gives an example of data captured in an Excel^®^ spreadsheet from a study on diabetes.^3^

**TABLE 1:** Example of data entered into an Excel^®^ spreadsheet.

Study code	Age	Sex	HbA1c	Chol	Wt	WC	DBP	SBP
BEVDH1	59	F	10.9	6.5	83.6	96.0	101.0	193.5
BEVDH2	61	F	-	-	82.5	118.0	79.0	157.5
BEVDH3	52	F	10.3	5.3	83.5	101.0	87.5	125.0
BEVDH4	56	F	11.0	7.1	75.0	108.0	93.0	135.5
BEVDH5	53	F	9.4	8.1	77.6	100.0	103.5	158.0
BEVDH6	55	F	5.1	6.7	92.3	108.0	116.0	186.0
BEVDH7	54	F	6.7	6.2	58.0	86.0	92.0	147.5
BEVDH8	68	F	7.8	4.0	81.5	104.0	71.5	118.5
BEVDH9	52	F	8.8	5.0	88.1	104.0	82.5	140.0
BEVDH10	68	M	9.4	4.3	107.0	121.0	87.0	173.5
BEVDH11	66	M	8.9	4.8	96.7	112.0	67.5	134.5
BEVDH12	72	F	6.2	6.1	81.0	97.0	80.5	136.5
BEVDH13	51	F	10.3	4.2	79.6	93.0	79.0	139.5
BEVDH14	46	M	6.3	5.3	62.1	81.0	98.5	156.0
BEVDH15	66	F	7.0	5.1	48.5	74.0	80.0	137.5
BEVDH16	64	F	8.7	5.7	68.4	95.0	79.0	145.5
BEVDH17	47	M	7.1	4.6	85.0	101.0	101.5	158.0
BEVDH18	64	M	5.9	5.6	75.0	102.0	86.0	132.5
BEVDH19	48	F	7.8	4.4	65.5	89.0	76.5	130.5
BEVDH20	55	F	6.9	7.6	61.3	86.0	101.5	154.0
BEVDH21	59	M	6.0	5.4	103.7	111.0	83.5	134.5
BEVDH22	28	F	12.3	3.4	59.0	80.0	81.0	104.5
BEVDH23	66	F	6.4	6.3	53.7	81.0	75.5	118.0
BEVDH24	48	F	9.8	5.1	92.5	94.0	99.5	140.5

HbA1c, Glycated haemoglobin; Chol, total cholesterol; Wt, weight; WC, waist circumference; DBP, diastolic blood pressure; SBP, systolic blood pressure.

Once all data are entered, they should be checked and cleaned. One way of doing this is to use the filter tool in Excel^®^ and look at the range of entries in each column. Obvious mistakes can then be recognised, such as numbers outside the expected range or which are clinically unlikely. Mistakes can then be addressed or cleaned up before analysing the data. This involves referring to the source document or questionnaire in order to recognise which information was entered incorrectly.

In your research proposal you should describe how you will capture, check and clean up your data. You should then describe the analytical software that you will be using and who may assist you, as well as the statistical tests that you expect to use. The rationale for selecting the various tests is outlined below.

### Data analysis

It is important to consider how many variables you want to analyse at the same time. ‘Univariate’ analysis involves only one variable, ‘bivariate’ analysis looks at the relationship between two variables and, when you have more than two variables, this is referred to as ‘multivariate’ analysis. The most common application of univariate analysis is in descriptive studies or when describing a sample that has been selected for a study. The most common application of bivariate or multivariate analysis is in observational or experimental studies where groups are being compared and the researcher wants to make inferences about any significant differences. Descriptive and inferential statistics are described below.

### Descriptive statistics

**FIGURE 1:**
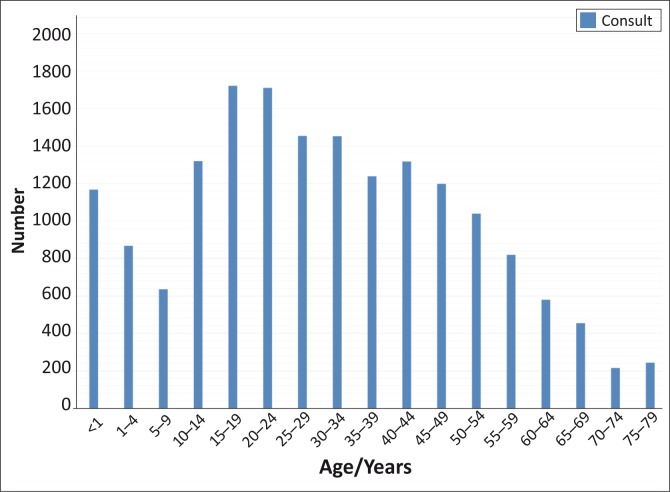
Example of a histogram showing the distribution of primary care consultations by age categories.

Categorical data can be analysed and presented as frequencies and percentages. These results can be described in the text, if very simple, such as the frequency and percentage of people that were male and female or, if more complicated, they can be presented in a table. When there are multiple categories, such as an age distribution, then a histogram can be helpful (see Figure 1).

Continuous data that are normally distributed can be analysed as a mean with a range, standard deviation or confidence interval. The standard deviation gives an idea of the spread of the total dataset from the mean (it is calculated as the square root of the variance). A small standard deviation implies that the data fall closely around the mean, whereas a large standard deviation implies that the data are more widely dispersed around the mean. The 95% confidence interval tells you that the real mean for the population is 95% likely to fall between the upper and lower limits of the interval. For purely descriptive purposes, the standard deviation would be preferred, whilst the confidence interval is more helpful when comparing data.

Continuous data that are not normally distributed can be analysed as a median with an interquartile range.

**FIGURE 2:**
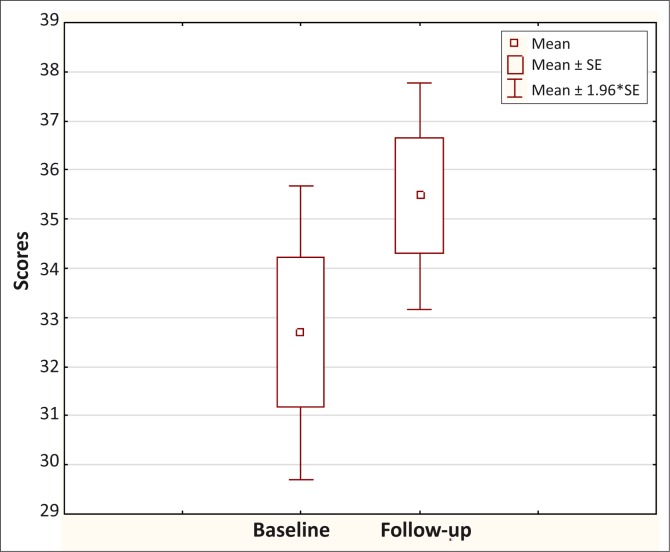
Example of a box-and-whisker plot for scores on the Kentucky Inventory of Mindfulness before and after a course on mindfulness-based stress interventions.

Data can be presented in text if very simple, such as the mean age with a standard deviation, or in a table, or in a histogram (normally distributed data) or a box and whisker plot (if not normally distributed). Table 2 gives an example from a study on diabetes of how such descriptive data can be presented in your research report, whilst Figures 1 and 2 give an example of a histogram and a box-and-whisker plot.

**TABLE 2:** Example of descriptive statistical results from a study on people with diabetes (*N* = 860).^3^

Results	*n* (%)
**Level of control**	
HbA1c < 7%	134 (15.6)
Cholesterol < 5.0 mmol/L	458 (53.3)
Diastolic BP < 80 mmHg	289 (33.6)
Systolic BP < 130 mmHg	345 (40.1)
Waist circumference –Women < 82 cm	40/650 (6.2)
Waist circumference – Men < 94 cm	80/209 (38.3)
**Clinical measurements**	**Mean (95% CI)**
HbA1c (%)	9.3 (9.2–9.5)
Cholesterol (mmol/L)	4.9 (4.8–5.0)
Weight (kg)	83.9 (82.7–85.2)
Waist circumference (cm)	100.3 (99.3–101.3)
Diastolic blood pressure (mmHg)	85.6 (84.7–86.5)
Systolic blood pressure (mmHg)	137.1 (135.4–138.7)
**Self-care activities**	**Mean (SD)**
Use of diet plan – days/week	4.7 (2.0)
Exercise – days/week	3.0 (2.3)
Foot care – days/week	5.6 (2.1)
Use of medication – days/week	6.9 (0.5)

HbA1c, Glycated haemoglobin; BP, blood pressure; CI, confidence interval; SD, standard deviation.

## Inferential statistics

Inferential statistics are used when you intend to compare groups and draw inferences about any statistically-significant differences. In broad terms, the following types of data can be compared:

- Continuous data versus categorical data.
- Categorical data versus categorical data.
- Continuous data versus continuous data.

In some comparisons, independent groups are compared with each other (i.e. two different groups), whilst in other situations, paired groups are compared over time (i.e. the same group at baseline and follow up). The following examples are based on the comparison of different independent groups.

### Continuous data versus categorical data

Analysis of variance (ANOVA) is used to determine whether the mean of the continuous variable differs between the different categories or groups. Again, different tests are used depending on whether the data is normally distributed (parametric tests such as *t*-test or *F*-test) or not normally distributed (non-parametric tests such as Mann-Whitney test for binary data, Kruskal-Wallis test for multiple categories). When in doubt, use a non-parametric test on the assumption that the data is not normally distributed. The analysis should give you a *p*-value that represents the likelihood of making a type 1 error (which infers that there is a difference, when in fact no difference really exists in the population). A statistically-significant *p*-value is usually set as less than 0.05 (less than a 5% chance of making a type 1 error).

**FIGURE 3:**
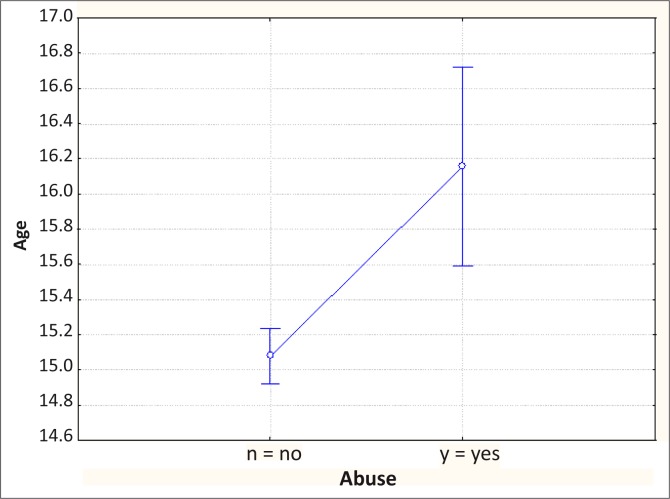
Example of analysis of variance (ANOVA) using a Mann-Whitney test to compare the age of high school students that use illicit substances with those that do not.

Figure 3 compares the mean age of high school students in Grades 8–10 who abuse illicit substances with those that do not.^4^ The graph shows the mean and 95% confidence intervals as well as the *p*-value derived from a Mann-Whitney test. This graph is produced by the analytical software and does not have to be copied in your results section when writing a research report. The *p*-value of < 0.01 in the example tells us that the two groups are significantly different. Table 3 demonstrates how these results could be presented in a table in your research report. One could also present this as the means and then the confidence interval for the difference between the means.

**TABLE 3:** Example of presentation of results shown in Figure 1 (*N* = 435).

Variables	Substance abuse *N* = 32 Mean (95% CI)	No substance abuse *N* = 403 Mean (95% CI)	*p*-value
**Age (years)**	16.2 (15.6-16.7)	15.1 (14.9–15.2)	< 0.01

CI, Confidence interval.

### Categorical data versus categorical data

The two sets of categories are compared in a contingency table (also called a cross-tabulation table or a ‘2 by 2’ table) and a chi-square test is used to determine whether there is any significant difference. When values in the cells of the table are very small (typically less than 5 or below 10 when there is only one degree of freedom), the Fisher Exact test is then used instead. Table 4 is an example of such a table, in which smoking habits are compared between high school students in Grades 8 and 10.^4^ The chi-square test gave a *p*-value of 0.772, indicating that there was no significant difference between smoking habits in Grade 8 when compared with Grade 10. Results can be presented in a table in your research report (Table 5) where the frequencies and percentages are compared and the *p*-value given to indicate whether there is any statistical difference.

**TABLE 4:** Example of a contingency (cross-tabulation) table.

Please insert heading	Non-smoker	Smoker	Row total
**Grade 8**	66	38	104
**Row%**	63.46%	36.54%	100.00%
**Grade 10**	74	47	121
**Row%**	61.16%	38.84%	100.00%
**Total**	140	85	225

**TABLE 5:** Example of presentation of results from analysis shown in Table 4.

Variables	Grade 8 students *N* = 104 *n* (%)	Grade 10 students *N* = 121 *n* (%)	*p*-value
**Tobacco smoking**	38 (36.5)	47 (38.8)	0.772

### Continuous data versus continuous data

Regression and correlation analysis are used to determine whether one continuous variable has a significant influence over or correlation with another continuous variable. This relationship can be plotted on a graph and if the regression line differs significantly from zero then there is a correlation. A correlation coefficient is usually calculated between 0 (no correlation) and ± 1 (complete correlation). The square of the correlation coefficient tells you what percentage of the dependent variable is determined by the variation of the independent variable being tested. These results are often best presented graphically, using a scatterplot and regression line.

**FIGURE 4:**
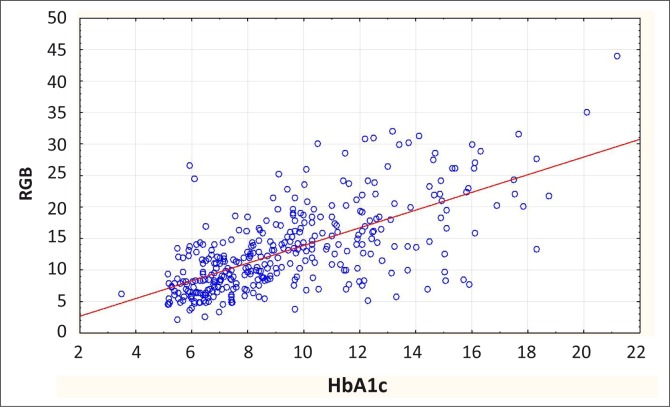
Example of a scatterplot and regression line for random blood glucose versus glycated haemoglobin values.

Figure 4 gives an example of such a scatterplot and regression line in which results for glycated haemoglobin (HbA1c) are correlated with random blood glucose results in the same group of diabetic patients.^5^ The correlation coefficient (*r*) was 0.67, indicating a moderate correlation and the square of the correlation (*r* ^2^) was 0.45, indicating that 45% of the variability of the random blood glucose can be explained by the HbA1c reading. The *p*-value was < 0.001, indicating that the relationship has statistical significance. This graph from the analysis is probably also the best way of presenting your results.

Clearly, the selection of a statistical test is not always easy and in many situations it is essential to consult a statistician. Table 6 gives a simplified guide to the commonly-used tests and comparisons.

**TABLE 6:** Selection of statistical tests for comparing data types between different groups in bivariate analysis.

Independent variable	Dependent variable			

	Categorical – binary	Categorical - nominal	Continuous – normally distributed	Continuous – non-normally distributed
**Categorical – binary**	Fisher exact†/chi square	Chi square	Independent samples *t*-test	Mann-Whitney test
**Categorical – nominal**	Chi square	Chi square	ANOVA	Kruskal-Wallis test
**Continuous – normally distributed**	Independent samples *t*-test	ANOVA	Pearson's correlation/linear regression	Spearman's correlation
**Continuous – non-normally distributed**	Mann-Whitney test	Kruskal-Wallis test	Spearman's correlation	Spearman's correlation

† Fisher exact test is used when one or more of the expected counts in a 2 x 2 table is small i.e. less than five.

### Presenting your results

The results section usually starts with the presentation of descriptive statistics that describe the sample or comparison groups. After this, inferential statistical results can be presented for the key comparisons that have been outlined in your objectives. Make use of subheadings to structure the presentation of your results.

It is important to stay aligned with your objectives and original intentions in performing your analysis and presenting your results. Do not start comparing everything with everything or performing more and more subgroup analyses in the hope of finding something significant. This ‘data dredging’ is frowned upon, because you are testing hypotheses that your study was never designed for.

Usually, the bulk of your results can be presented most clearly in a few well-designed tables (see Tables 3 and 4). You will usually have to extract the relevant results from your analysis document and re-organise them in tables designed for this purpose. It is seldom possible to just copy and paste tables from your analysis documents. Do not present endless figures (e.g. histograms, pie charts) for each variable and do not present the same results in both tables and figures unless there is a specific reason to do so. For example, you may want to highlight an important result that is more obvious when shown in a figure (see Figure 4). Take care when cutting and pasting graphs from your analytical software package as they may need editing and formatting in order to be clear to another reader.

Once you have constructed your tables and any additional figures you will need to provide the text to go with them. Each table and figure should be numbered sequentially and given a title. Tables are traditionally numbered with Roman numerals (e.g. I, II, III, IV) and the title placed above the table, although it is always important to consult the house style when converting from a thesis to an article, as the requirements may change, depending on the publication. Figures, on the other hand, use Arabic numbers (e.g. 1, 2, 3, 4) and the title is placed below the figure. Always take care when formulating the titles and legends of your figures and tables. Many readers will only have a look at the figures and tables, without reading your text. They should be able to pick up and understand the most important messages on the basis of title, figures and tables only.

It is usual to introduce and refer to each table beforehand in the text, in order to make sure your reader knows what the table is about and how to make sense of it, especially if there is anything that needs additional explanation. You can provide some interpretation of the results for the reader, without discussing them more broadly, for example, by highlighting the most significant findings. However do not be tempted to repeat all the results in the text. If there are any additional related results that are not in the table, these can also be given in the text.

## Conclusion

Researchers should identify the type of quantitative data they will be collecting and ensure that they are captured cleanly and accurately in a spreadsheet. The data can then be analysed using any of the commercially-available statistical software. Descriptive statistics can be presented as frequencies with percentages, means with standard deviations or confidence intervals, or as medians with an interquartile range. Inferential statistics can compare continuous and categorical data types so as to determine whether there is a statistically-significant difference using a variety of carefully-selected statistical tests. The final results should be presented in tables, supported by the text and, if necessary, additional figures.
